# Inattention Predicts Increased Thickness of Left Occipital Cortex in Men with Attention-Deficit/Hyperactivity Disorder

**DOI:** 10.3389/fpsyt.2017.00170

**Published:** 2017-09-13

**Authors:** Peter Sörös, Katharina Bachmann, Alexandra P. Lam, Manuela Kanat, Eliza Hoxhaj, Swantje Matthies, Bernd Feige, Helge H. O. Müller, Christiane Thiel, Alexandra Philipsen

**Affiliations:** ^1^School of Medicine and Health Sciences, Psychiatry and Psychotherapy, University Hospital Karl-Jaspers-Klinik, University of Oldenburg, Oldenburg, Germany; ^2^Research Center Neurosensory Science, University of Oldenburg, Oldenburg, Germany; ^3^Department of Psychology, Biological Psychology Lab, School of Medicine and Health Sciences, University of Oldenburg, Oldenburg, Germany; ^4^Department of Psychology, Laboratory for Biological Psychology, University of Freiburg, Freiburg, Germany; ^5^Faculty of Medicine, Department of Psychiatry and Psychotherapy, Medical Center – University of Freiburg, Freiburg, Germany

**Keywords:** attention-deficit/hyperactivity disorder, attention, cortical thickness, occipital cortex, morphometry, gray matter, FreeSurfer

## Abstract

**Background:**

Attention-deficit/hyperactivity disorder (ADHD) in adulthood is a serious and frequent psychiatric disorder with the core symptoms inattention, impulsivity, and hyperactivity. The principal aim of this study was to investigate associations between brain morphology, i.e., cortical thickness and volumes of subcortical gray matter, and individual symptom severity in adult ADHD.

**Methods:**

Surface-based brain morphometry was performed in 35 women and 29 men with ADHD using FreeSurfer. Linear regressions were calculated between cortical thickness and the volumes of subcortical gray matter and the inattention, hyperactivity, and impulsivity subscales of the Conners Adult ADHD Rating Scales (CAARS). Two separate analyses were performed. For the first analysis, age was included as additional regressor. For the second analysis, both age and severity of depression were included as additional regressors. Study participants were recruited between June 2012 and January 2014.

**Results:**

Linear regression identified an area in the left occipital cortex of men, covering parts of the middle occipital sulcus and gyrus, in which the score on the CAARS inattention subscale predicted increased mean cortical thickness [*F*(1,27) = 26.27, *p* < 0.001, adjusted *R*^2^ = 0.4744]. No significant associations were found between cortical thickness and the scores on CAARS subscales in women. No significant associations were found between the volumes of subcortical gray matter and the scores on CAARS subscales, neither in men nor in women. These results remained stable when severity of depression was included as additional regressor, together with age.

**Conclusion:**

Increased cortical thickness in the left occipital cortex may represent a mechanism to compensate for dysfunctional attentional networks in male adult ADHD patients.

## Introduction

Attention-deficit/hyperactivity disorder (ADHD) is a serious and frequent psychiatric disorder in children, adolescents and adults with the core symptoms inattention, impulsivity, and hyperactivity (1). Several lines of evidence have established a strong neurobiological background of ADHD. Today, ADHD is seen as a disorder of multiple neural systems mainly subserving sensory and cognitive functions as well as of the default mode network (2).

### Structural Brain Imaging in Children and Adolescents with ADHD

In numerous cross-sectional studies, MRI has been used to identify local and global differences in brain structure and function between individuals with ADHD and age-matched healthy participants (3, 4). In early and influential studies on boys with ADHD, significantly smaller volumes of the left globus pallidus (5) and left caudate nucleus (6) were found. The study by Filipek et al. was the first to detect significantly smaller volumes of the right frontal area and the bilateral retrocallosal parietal–occipital region, thus supporting the hypothesis of dysfunctional frontoparietal circuits in ADHD (6).

As the available studies on brain morphology in ADHD have pointed at gray matter reductions in several different areas, quantitative meta-analyses of voxel-based morphometry data have been performed to identify changes across a larger number of studies and patients. Based on seven original studies, the quantitative meta-analysis of Frodl et al. has found significantly smaller volumes of the right globus pallidus and right putamen in children and adolescents with ADHD compared to healthy individuals, but no cortical differences (7). In a more recent quantitative meta-analysis, a subgroup-analysis of 17 pediatric studies has demonstrated a significantly smaller gray matter volume in a cluster including the right putamen, pallidum, and insula, in both left and right caudate nuclei, right cerebellum, and a cluster including the right anterior cingulate cortex and ventromedial orbitofrontal cortex (8).

### Structural Brain Imaging in Adults with ADHD

In adult patients with ADHD, subcortical abnormalities were also detected, such as smaller volumes of the right (9) and left caudate nucleus (10) as well as smaller volumes of the bilateral amygdalae (11), the right thalamus, and the bilateral cerebellar hemispheres (12). Moreover, a global reduction of cortical thickness was found in adult ADHD (13). Localized reductions of cortical thickness were identified in the right frontal lobe (14), the right parietal lobe (15), and the bilateral visual cortices (16). Thinning of the anterior cingulate cortex (15) and a volume reduction of the anterior cingulate gyrus (17) were also reported. Cubillo et al. conclude in their review that the structural and functional abnormalities seen in children and adolescents with ADHD in fronto-cortical and fronto-subcortical networks persist into adulthood (18).

By contrast, a large study on 131 adult patients with ADHD and 95 age-matched healthy individuals did not find local changes in gray matter volume between groups (19). The quantitative meta-analysis by Frodl et al. found only a significant reduction of anterior cingulate volume in adult ADHD compared to healthy controls (7). A more recent quantitative meta-analysis reported significantly decreased gray matter volume in the ventromedial orbitofrontal cortex, the right posterior cingulate cortex, and the right putamen of adult patients with ADHD (8). Finally, a large multicenter study performed by the ENIGMA working group did not find significant differences in subcortical gray matter volumes between approximately 500 adult patients with ADHD 21 years of age or older and approximately 400 healthy controls (20), comparing the volumes of the accumbens nucleus, amygdala, caudate nucleus, hippocampus, pallidum, putamen, and thalamus.

To summarize, the existing literature on morphological characteristics of pediatric and adult ADHD provides an inconsistent and in parts contradictory picture of the neuroanatomical basis of ADHD. The remarkable differences between morphometric studies of ADHD and the small number of commonalities are probably due to several factors. First, ADHD is a remarkably heterogeneous disorder. Individual patients have distinct combinations of ADHD symptoms and comorbidities, each at an individual level of severity. A small number of studies have taken this heterogeneity into account, reporting significant correlations between disease severity and cortical thickness (14) or the volume of the right caudate nucleus (9). Second, the brains of girls and boys as well as women and men with ADHD may be affected in a differential way (21, 22). A study on the distinct morphology of the frontal lobe in 64 boys and 29 girls with ADHD supports this notion (23). As boys and men are overrepresented in most structural brain studies in ADHD, it remains questionable if their results also apply to girls or women.

### The Present Study

The objective of the present study was to investigate potential associations between the severity of the characteristic symptoms of adult ADHD—inattention, hyperactivity, and impulsivity—and (a) cortical thickness and (b) subcortical gray matter volumes for all patients and for men and women separately. We hypothesized that more severe ADHD symptoms will be associated with decreased cortical thickness and smaller subcortical gray matter volumes in regions involved in the control of the respective brain functions (9, 14).

## Materials and Methods

### Participants

The data of 64 ADHD patients (35 women, 29 men) were analyzed. The clinical characteristics of all participants are summarized in Table 1. This study is part of a larger randomized controlled trial registered in the ISRCTN registry (ISRCTN12722296)^1^ and approved by the Ethics Committee of the Faculty of Medicine, University of Freiburg, Germany. As this clinical trial consisted of a treatment arm (ADHD patients who performed mindfulness meditation) and an active control arm (ADHD patients who received psychoeducation), no healthy control group was included. Structural data were acquired prior to randomization of patients into the control/treatment arm.

**Table 1:** Demographics.

	All patients (*n* = 64)	Women (*n* = 35)	Men (*n* = 29)	*p*-Value
Age (years)	40.2 ± 11.0 (19–61)	42.1 ± 10.7 (21–61)	37.9 ± 11.0 (19–56)	0.1329
Attention-deficit/hyperactivity disorder (combined subtype)	53 (82.8%)	30 (85.7%)	23 (79.3%)	0.4101
ADD (inattentive subtype)	11 (17.2%)	5 (14.3%)	6 (20.7%)	1
**Conners Adult ADHD Rating Scales subscales**				
Inattention	21.0 ± 7.2	23.4 ± 5.2	18.2 ± 8.2	0.005195
Hyperactivity	17.4 ± 7.7	19.0 ± 7.7	15.4 ± 7.3	0.06593
Impulsivity	17.4 ± 8.6	20.4 ± 7.9	13.8 ± 8.0	0.001707
**Comorbidities**				
Depression	37 (57.8%)	23 (65.7%)	14 (48.3%)	0.1877
Beck Depression Inventory-II score	16.1 ± 10.6	18.2 ± 8.1	13.6 ± 12.6	0.0952
Anxiety disorder	11 (17.2%)	5 (14.3%)	6 (20.7%)	1
Personality disorder	6 (9.4%)	3 (8.6%)	3 (10.3%)	1
Obsessive–compulsive disorder	2 (3.1%)	1 (2.9%)	1 (3.4%)	1
**Education**				0.7814
Secondary school (until grade 9)^a^	9 (14.1%)	4 (11.4%)	5 (17.2%)	
Secondary school (until grade 10)^b^	22 (34.4%)	11 (31.4%)	11 (37.9%)	
High school diploma^c^	21 (32.8%)	13 (37.1%)	8 (27.6%)	
University degree	12 (18.8%)	7 (20.0%)	5 (17.2%)	
**Employment**				0.126
In education	15 (23.4%)	5 (14.3%)	10 (34.5%)	
Employed	40 (62.5%)	23 (65.7%)	17 (58.6%)	
Unemployed	3 (4.7%)	3 (8.6%)	0	
Retired	6 (9.4%)	4 (11.4%)	2 (6.9%)	
**Monthly income**				0.02552
No answer	11 (17.2%)	6 (17.1%)	5 (17.2%)	
≤1,500 €	35 (54.7%)	23 (65.7%)	12 (41.4%)	
1,501–3,000 €	14 (21.9%)	6 (17.1%)	8 (27.6%)	
>3,000 €	4 (6.3%)	0	4 (13.8%)	

*Data are presented as mean ± SD. The *p* values refer to Welch’s *t*-test (for continuous data) and to the exact binomial test or Pearson’s chi-squared test (for discrete data) comparing women and men*.

*^a^Hauptschulabschluss*.

*^b^Mittlere Reife*.

*^c^Abitur*.

Participants were recruited from the specialized ADHD in adulthood outpatient clinic as well as from the inpatient units of the Department of Psychiatry and Psychotherapy, Medical Center—University of Freiburg, Germany. In addition, the study was announced on the website of the University Hospital, the local ADHD support group and on the website of the German ADHD advocacy organization.^2^ All participants provided written informed consent.

Inclusion criteria were as follows: age between 18 and 65 years, diagnosed with ADHD, free from ADHD medication or any psychotherapeutic treatment during study participation, and verbal IQ > 85. Exclusion criteria were as follows: previous therapy with methylphenidate (even during childhood), a comorbid diagnosis of schizophrenia, bipolar disorder type I, antisocial or borderline personality disorder, drug addiction, developmental disorders (e.g., autism), acute suicidal ideations, auto-aggressive behavior and neurological disorders, as well as MRI-specific contraindications (e.g., metal implants).

### Diagnosis, Clinical Data, and Socioeconomic Data

The diagnosis of ADHD was established by expert psychiatrists according to DSM-IV criteria (24) and validated using rating scales, including the German version of the Wender Utah Rating Scale for the retrospective assessment of childhood ADHD symptoms (25) and the Conners Adult ADHD Rating Scales-Self Report as a measure of ADHD symptom severity in adulthood (26) in the German long version with 66 items (27). For this study, the scores on the inattention/memory problems, hyperactivity/restlessness, and impulsivity/emotional lability subscales were used as regressors in the analysis of cortical thickness and subcortical gray matter volume. Each subscale contains 12 unique items, which were graded by the patient on a 4-level Likert scale (0 for “not at all or never,” 1 for “just a little, once in a while,” 2 for “pretty much often,” and 3 for “very much, very frequently”).

Participants were evaluated for comorbidity using the German version of the Structured Clinical Interview for DSM-IV (SKID) (28). The German version of the Choice Verbal Intelligence Test (MWT-B) was used to measure verbal IQ (29). The German version of the Beck Depression Inventory-II (BDI-II), a 21-item self-report questionnaire, was administered to assess the severity of depression (30). To evaluate the socioeconomic status, patients were asked for information about their education, employment status, and monthly income.

### MRI Data Acquisition

T1-weighted anatomical images of the brain were acquired on a 3-T Siemens Magnetom Trio with a 12-channel head coil at the Freiburg Brain Imaging Center using a three-dimensional magnetization-prepared rapid acquisition gradient echo sequence with the following parameters: voxel size 1 mm × 1 mm × 1 mm, field of view = 256 mm, 256 × 256 matrix, 160 sagittal slices, slice thickness 1 mm, phase encoding direction anterior → posterior, repetition time (TR) = 2,200 ms, echo time (TE) = 4.11 ms, non-selective inversion recovery, inversion time (TI) = 1,000 ms, flip angle = 12°, no acceleration, and acquisition time 7:04 min.

### Analysis of Individual Structural MRI Data

Cortical reconstruction and volumetric segmentation were performed with the FreeSurfer 5.3 image analysis suite on Mac OS X (31).^3^ Image processing included removal of non-brain tissue using a hybrid watershed and surface deformation procedure, intensity normalization, and segmentation of the subcortical white matter and deep gray matter structures (including caudate nucleus, putamen, pallidum, hippocampus, amygdala, and accumbens nucleus) (32, 33). The gray–white and gray–cerebrospinal fluid (pial) surfaces were modeled as a triangular mesh and were placed at the location where the greatest shift in intensity defines the transition to the other tissue class (34, 35), followed by automated topology correction. Cortical thickness was calculated as the closest distance from the gray–white to the pial surface at each vertex on the tessellated surface (35).

The thickness maps are not restricted to the voxel resolution of the original data (i.e., 1 mm × 1 mm × 1 mm) and thus can detect submillimeter differences between groups. Measurements of cortical thickness using FreeSurfer have been validated against histological analysis (36) and manual measurements (37). The cerebral cortex was parcellated into units with respect to gyral and sulcal structure, based on the cortical atlas by Destrieux et al. (38). For quality control, reconstructed surfaces and subcortical segmentations were inspected for structural abnormalities, accuracy of registration, and presence of artifacts. One of originally 65 patients was excluded because the surface reconstruction resulted in a gyral defect. The surface reconstructions of the remaining 64 participants were accurate and could be used without further manual edits.

### Group Analysis of Structural MRI Data

For group analysis, the cortical thickness maps of all individual participants were resampled onto the fsaverage [Montreal Neurological Institute (MNI) 305] template and spatially smoothed at 10 mm full with at half maximum (using FreeSurfer’s *mris_preproc* and *mri_surf2surf* commands). A linear model was used to calculate the effect of the scores on the CAARS inattention, hyperactivity, and impulsivity subscales on cortical thickness (using FreeSurfer’s *mri_glmfit*) for all participants and for women and men separately.

To account for the decrease of cortical thickness and the loss of subcortical gray matter volume during healthy aging, the age of each participant was added to every regression model. Research on healthy individuals between 18 and 87 years of age has shown a linear decrease of mean cortical thickness across the entire age span (39). In a second group analysis, the BDI-II score was used as additional regressor, together with age.

Cluster-wise correction for multiple comparisons was performed by running a Monte Carlo simulation (using FreeSurfer’s *mri_glmfit-sim* command with 10,000 simulations and a vertex-wise cluster-forming threshold of *p* < 0.01).^4^

**Figure 1:**
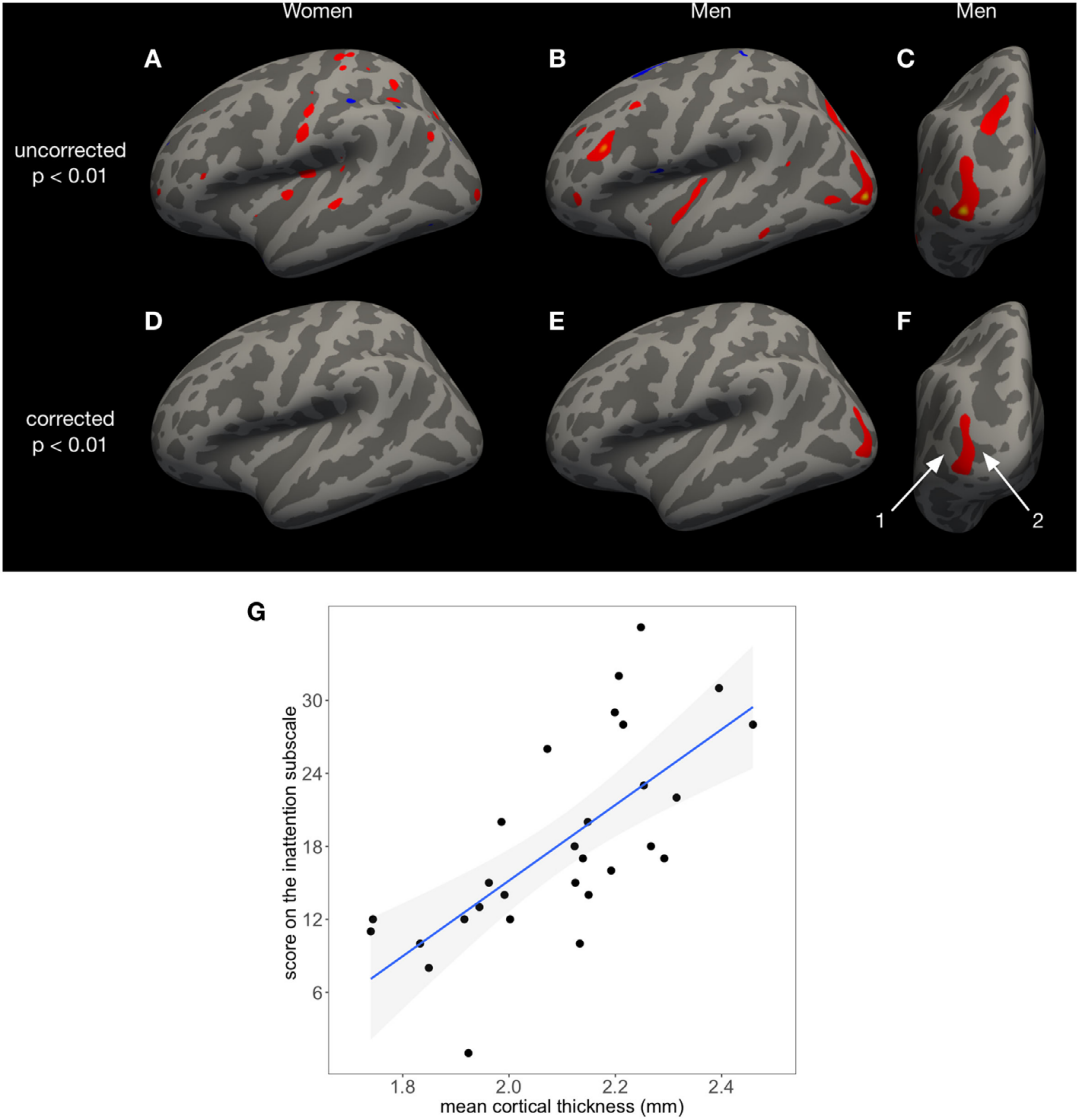
The score on the Conners Adult ADHD Rating Scales inattention subscale predicts cortical thickness in a left occipital area in men, covering parts of the middle occipital gyrus (1) and sulcus (2), but not in women. The uncorrected results of FreeSurfer’s surface-based analysis (*p* < 0.01) are shown in panel **(A)** for women and panels **(B,C)** for men. After cluster-wise correction for multiple comparisons, only the left occipital area in men remains significant [*p* < 0.01 **(E,F)**]. No significant associations between cortical thickness and inattention were found in women **(D)**. In panel **(G)**, individual mean cortical thickness in the left occipital area seen in panels **(E,F)** is shown as a function of the inattention score. Regression line and 95% confidence intervals (gray shade) are displayed [*F*(1,27) = 26.27, *p* < 0.001, adjusted *R*^2^ = 0.4744].

The mean cortical thickness in the significant occipital cluster (Figure 1) was retrieved for each patient from FreeSurfer’s *y.ocn.dat*. The mean cortical thickness was linearly regressed against the individual score on the inattention subscale using the statistical package R on Mac OS X and R’s *lm* function.^5^ To estimate the effect size, the adjusted *R*^2^ was calculated. For an improved visualization of the location and extent of the significant left occipital cluster, 125 movie frames were saved using FreeSurfer’s *Freeview* visualization tool, starting from a left lateral view of the brain and advancing 1° per frame in clockwise direction. The frames were then converted to a Quicktime movie with 25 frames per second using Adobe Photoshop CS4 and the H.264 video compression standard (see Video S1 in Supplementary Material).

In addition, CAARS scores were linearly regressed against the volumes of the subcortical structures as determined by Freesufer’s subcortical segmentation using R for all participants and for women and men separately. In the first group analysis, age was used as additional regressor. In the second group analysis, age and the BDI-II score were both used as additional regressors.

For an analysis of age-related morphometric changes, cortical thickness and subcortical gray matter volume were regressed against age (Figure 3; Table 3).

To test for sex-specific effects, a linear regression was performed to compare cortical thickness and subcortical gray matter volume between women and men. To account for differences in brain size between the sexes, total intracranial volume, as assessed by FreeSurfer, was used as regressor together with age (40).

### Analysis of Clinical Data

**Figure 2:**
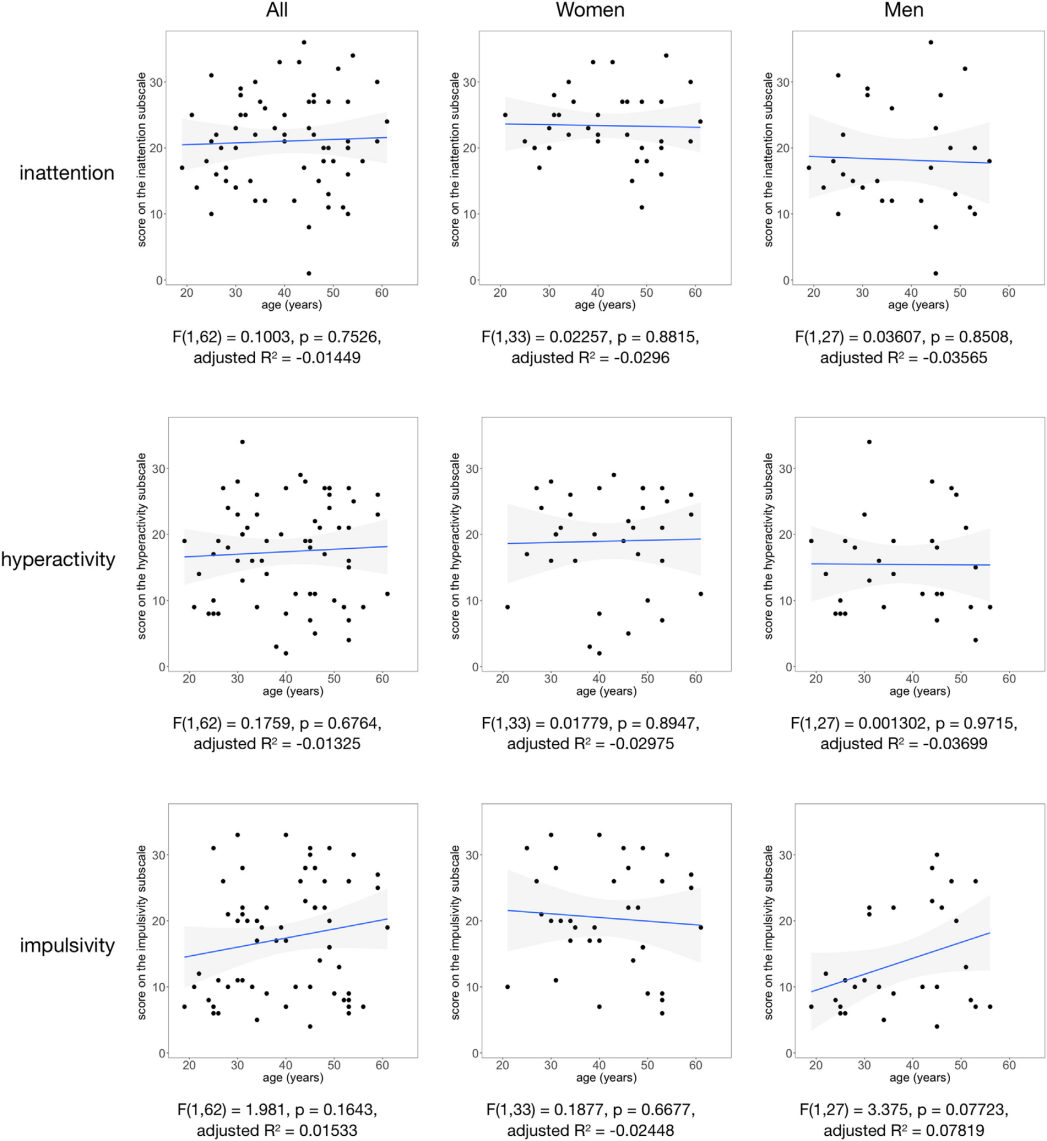
The scores on the Conners Adult ADHD Rating Scales inattention, hyperactivity, and impulsivity subscales are shown as a function of age for all participants (left column), women (middle column), and men (right column). Regression line and 95% confidence intervals (gray shade) are displayed.

Welch’s two-sample *t*-test was calculated to compare the mean age and CAARS scores between men and women using R (Table 1). The exact binomial test was used to compare discrete data between men and women (ADHD, ADD, depression, personality disorder, and obsessive–compulsive disorder; Table 1). Pearson’s chi-squared test was used to compare the frequencies of the different categories for education, employment, and monthly income (Table 1). To test for associations between ADHD symptom severity and age, CAARS scores were linearly regressed against age (Figure 2).

## Results

### Demographics

Table 1 summarizes age and CAARS scores in the entire sample (*n* = 64) and in women and men separately. Women had significantly higher scores on the inattention (*p* < 0.01) and impulsivity subscales (*p* < 0.01) than men. The most frequent comorbidities were depression and anxiety disorder. Comorbid depression was diagnosed in 23 of 35 women (65.7%) and in 14 of 29 men (48.3%). The BDI-II score was 19 or higher (indicating at least moderate depression) in 27 of 64 patients (42.2%).

Education and employment status were similar in women and men with the majority of patients currently in an education program or regularly employed: 28 of 35 women (80%) and 27 of 29 men (93%). There was a significant difference in personal monthly income with more women in the lower income group. This difference has to be interpreted with caution because 11 patients (17.2%) chose not to answer this question.

### Association between CAARS Scores and Cortical Thickness

Using linear regression, we found an area in the left occipital cortex of men, covering parts of the middle occipital gyrus and sulcus, in which higher scores on the CAARS inattention subscale predicted increased mean cortical thickness [*F*(1,27) = 26.27, *p* < 0.001, adjusted *R*^2^ = 0.4744, Figure 1]. The MNI coordinates for the vertex of highest significance were in the left middle occipital gyrus (*x* = −35.7, *y* = −85.2, and *z* = −1.4; corrected *p* = 0.01375). For visualization purposes, the pial surface was inflated in Figures 1A–F (41). A movie shows the exact location and extent of this significant cluster (Video S1 in Supplementary Material).

For Figure 1G, the mean cortical thickness in the left occipital area shown in red in Figures 1E,F was calculated for each male participant and was then plotted against the individual score on the inattention subscale. In women, inattention was not significantly associated with cortical thickness (Figure 1D). Both in women and men, hyperactivity and impulsivity were not associated with cortical thickness.

Adding the BDI-II depression scores as additional regressor, together with age, to the regression models did not change the overall results. The left occipital area in men remained significant. Additional significant clusters did not emerge.

### Association between CAARS Scores and Subcortical Gray Matter Volumes

Significant associations between subcortical gray matter volumes and CAARS scores were found in linear regression models with age as additional regressor (Appendix S1 in Supplementary Material). Among the associations with highest *p* values were a positive relationship between the volume of the left pallidum and the CAARS inattention score and a negative relationship between the volume of the left hippocampus and the CAARS impulsivity score, both in women. After Bonferroni correction for multiple comparisons, these associations were not significant anymore (corrected *p* < 0.05/108 = 0.0004).

Again, adding the BDI-II depression scores as additional regressor, together with age, to the regression models did not change the overall results (Appendix S2 in Supplementary Material). After Bonferroni correction for multiple comparisons, no significant associations between CAARS scores and subcortical gray matter volume remained.

### Association between Severity of Depression and Cortical Thickness or Subcortical Gray Matter Volumes

Linear regression did not reveal a significant association between severity of depression, as represented by BDI-II scores, and cortical thickness in all patients. Similarly, no significant association was found between severity of depression and volumes of subcortical gray matter.

### Association between CAARS Scores and Age

No significant associations were found between CAARS scores and age using linear regression (Figure 2). Moreover, there was no significant interaction effect between CAARS scores and age in multiple linear regression (Appendix S1 in Supplementary Material).

### Association between Age and Cortical Thickness

**Figure 3:**
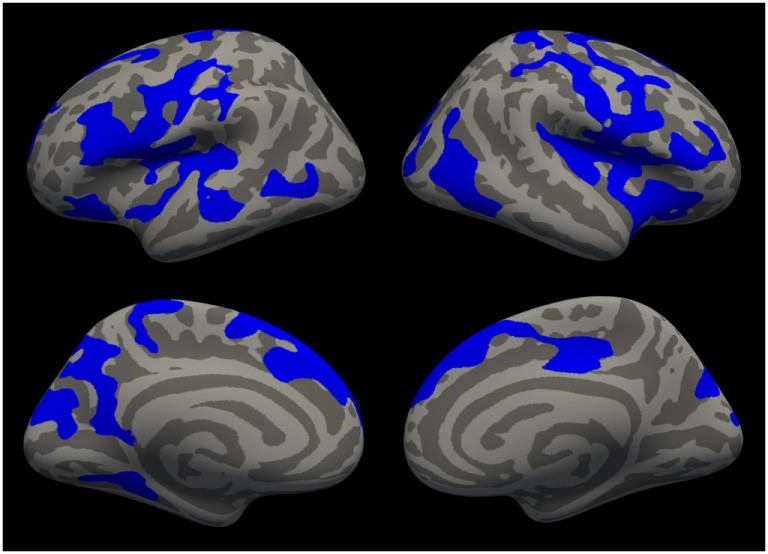
Cortical areas in which cortical thickness significantly decreases with age are shown in blue after cluster-wise correction for multiple comparisons (*p* < 0.01) for all patients (*n* = 64).

Figure 3 illustrates brain regions in which cortical thickness significantly decreases with age after cluster-wise correction for multiple comparisons (surface-based analysis of all participants, *n* = 64). Table 2 summarizes the significant clusters in the left and right hemispheres shown in Figure 3.

**Table 2:** Age-related decrease of cortical thickness.

Hemisphere	Region	*x* (mm)	*y* (mm)	*z* (mm)	Size (mm^2^)	*p*-Value
Left	Pars opercularis	−51	13.7	14.5	7,238.17	<0.001
Left	Superior frontal	−6.2	44.3	39.6	3,781.02	<0.001
Left	Precuneus	−8.7	−58.4	12.3	1,667.50	<0.001
Left	Postcentral	−7.7	−39.1	69.5	1,445.48	<0.001
Left	Cuneus	−3.8	−76.8	20.8	1,243.02	<0.001
Left	Fusiform	−35	−44.4	−14	791.85	<0.001
Left	Lateral orbitofrontal	−40	25.9	−13	757.29	0.001
Left	Inferior temporal	−51	−63.1	−3	621.06	0.00459
Left	Superior parietal	−26	−40	51.6	538.25	0.01077
Left	Rostral middle frontal	−39	47.4	8.2	516.72	0.01395
Right	Lateral orbitofrontal	29.4	24.3	−0.7	9,523.02	<0.001
Right	Superior frontal	7.6	4.4	54.6	2,498.33	<0.001
Right	Inferior parietal	43.3	−64.3	6.3	1,721.20	<0.001
Right	Superior parietal	18.2	−86.3	20.3	1,391.03	<0.001
Right	Postcentral	17.6	−36	67.7	617.76	0.0038
Right	Superior parietal	11.5	−81.8	33.4	600.39	0.00459
Right	Precuneus	11.8	−46.8	65.9	518.42	0.01296
Right	Superior parietal	30.4	−55.6	42	510.94	0.01355

*The table lists all clusters in which cortical thickness significantly decreases with age. Size and p-value of each cluster are noted. The anatomical location of the vertex with highest significance and its x, y, and z coordinates in Montreal Neurological Institute 305 space are given*.

### Association between Age and Subcortical Gray Matter Volumes

Table 3 summarizes the associations between age and subcortical gray matter volumes for all patients (*n* = 64). After Bonferroni correction for multiple comparisons, the volumes of left and right putamen, left and right pallidum, left and right accumbens, and right caudate were significantly associated with age at a corrected *p* < 0.05/12 = 0.004. The volumes of left and right hippocampus as well as left and right amygdala were not associated with age, even at an uncorrected *p* < 0.05.

**Table 3:** Age-related decrease of subcortical gray matter volumes.

	*F*(1,62)	*p*-Value
Left caudate	7.931	0.00651**
Left putamen	15.49	<0.001***
Left pallidum	10.74	0.00172**
Left hippocampus	2.535	0.116
Left amygdala	1.254	0.267
Left accumbens	10.91	0.00159**
Right caudate	11.12	0.00145**
Right putamen	23.62	<0.001***
Right pallidum	15.98	<0.001***
Right hippocampus	1.909	0.172
Right amygdala	2.363	0.129
Right accumbens	10.35	0.00206**

*The table lists the F-statistics and p values of the linear regression model*.

*lm(volume ~ age) for all patients*.

**Uncorrected p < 0.01.

***p < 0.001.

### Sex Differences in Cortical Thickness or Subcortical Gray Matter Volumes

Cortical thickness was not significantly different between men and women when using age and total intracranial volume as additional regressors, after cluster-wise correction for multiple comparisons.

Moreover, the volumes of subcortical gray matter were not significantly different between men and women when using age and total intracranial volume as additional regressors, after Bonferroni correction.

## Discussion

The present study performed surface-based morphometry in a group of 64 adult patients with ADHD, 35 women and 29 men, who have never been treated with methylphenidate. In men, but not in women, linear regression identified an area covering parts of the left middle occipital sulcus and gyrus, in which the individual score on the CAARS inattention subscale predicted increased cortical thickness: men suffering from more severe inattention had higher mean cortical thickness in this area (Figure 1). No further associations between CAARS subscale scores and cortical thickness were found, neither in men nor in women. Moreover, the study could not identify significant associations between one of the CAARS subscales and subcortical gray matter volumes, neither in men nor in women. Our results were not confounded by the severity of depression.

### Positive Relationship between Cortical Thickness in Middle Occipital Gyrus and Inattention

Even though frontostriatal networks have often been reported with respect to functional and structural abnormalities in ADHD, there is increasing evidence for structural and functional changes in occipital regions (16) with the left middle occipital gyrus being consistently found across several studies (42). A prior study found increased gray matter volume in the left middle occipital gyrus in adult ADHD patients as compared to control subjects (43). Our evidence complements these findings in showing that gray matter increases are related to self-reported inattention in male ADHD patients and are thus of behavioral relevance. The left middle occipital gyrus in ADHD patients also exhibits changes in functional and structural connectivity. Diffusivity reductions in this region were reported in a sample of adult unmedicated ADHD patients (44). In children with ADHD, the left middle occipital gyrus was found to show reduced functional connectivity (45). Some studies related measures of structural connectivity in occipital regions to attentional deficits and provided evidence that the inattentive subtype in children is related to increased radial diffusivity in temporo-occipital areas and lower fractional anisotropy in the inferior frontal–occipital fasciculus which connects frontal and occipital cortices and may thus modulate visual information processing and attention (46). The middle occipital gyrus is further functionally connected with the dopaminergic ventral tegmental area/substantia nigra, and this negative connectivity is reduced with acute methylphenidate in healthy volunteers (47).

We therefore suggest that the left middle occipital gyrus represents an important node in attentional networks and speculate that increased cortical thickness in the left middle occipital gyrus in adult ADHD patients may represent a mechanism to compensate for dysfunctional attentional networks (48), most likely through increased dendritic branching and number of synapses (49). Whether this mechanism is specific to male brains needs to be confirmed in future studies with larger sample sizes. Several investigators have suggested increased gray matter in individuals with specialized training, such as in the hippocampus of taxi drivers (50), the parietal cortex of mathematicians (51), the primary sensorimotor cortex and cerebellum of pianists (52), and the orbitofrontal cortex and hippocampus in active meditators (53). Although most morphometric studies in ADHD reported decreased gray matter in patients with ADHD, there is also evidence for areas of increased gray matter. The first study that suggested areas of increased cortical thickness in patients with ADHD compared 21 children, 18 adolescents, and 20 adults with ADHD with healthy age-matched controls (54). In children and adolescents, the authors observed areas of increased cortical thickness mainly in the right medial and lateral occipital cortex and in the right temporal cortex, which seemed to disappear in adulthood (54).

Our understanding of anatomical and functional brain differences between male and female patients with ADHD is still in its infancy. Only a small number of studies have covered this topic so far, most of them with small sample sizes. At present, there is no well-founded explanation why cortical thickness in the left occipital cortex is associated with inattention in the men, but not in the women of our sample.

### No Negative Relationships between Cortical Thickness and Subcortical Volumes and Symptom Severity

In our sample of adult ADHD patients, no cortical or subcortical regions were identified in which higher scores on the CAARS subscale were associated with decreased cortical thickness or subcortical gray matter volume. Thus, we did not find support for our initial hypothesis, predicting cortical thinning in patients with more severe ADHD core symptoms. Our results provide additional evidence for the notion that morphometric alterations seen in children and adolescents with ADHD may diminish or even disappear during the transition into adulthood (20, 55).

### No Association between the Severity of Depression and Cortical Thickness or Subcortical Gray Matter Volumes

Recent studies have shown increased cortical thickness in patients with first-episode depression (but without ADHD), primarily in the frontal cortex and cingulate gyrus (56, 57). Large meta-analyses have indicated that patients with major depressive disorder have reductions of gray matter volume, mainly in the anterior cingulate and orbitofrontal cortex (58, 59), and of hippocampal volume (60, 61).

Depression is the most frequent comorbidity in adult ADHD (62, 63). Adult patients with ADHD and comorbid major depressive disorder were found to have smaller hippocampal volumes than ADHD patients without depression (64). In our exploratory analysis, the severity of depression was not associated with morphometric changes in adult ADHD.

### Age-Related Decrease in Cortical Thickness and Subcortical Gray Matter Volumes

In another exploratory analysis of our cross-sectional sample, we found that age is associated with widespread cortical thinning and volume reduction of the basal ganglia (Figure 3; Tables 2 and 3), similar to the pattern of atrophy found in healthy elderly (39, 65, 66). Future longitudinal studies are warranted to directly compare age-related cortical and subcortical decline in ADHD patients with healthy aging.

### No Association between Sex and Cortical Thickness or Subcortical Gray Matter Volumes

In healthy adults, the morphology of female and male brains differs in various respects, mainly in areas related to the limbic and language systems (67). Specifically, women show regions of increased cortical thickness compared to men (68, 69).

In boys with ADHD, gray matter volumes of the ventral anterior cingulate cortex were decreased compared to healthy boys and increased in girls with ADHD compared to healthy girls (70). In men, but not women, with ADHD, the volume of the right caudate was smaller compared to healthy controls (64).

In our exploratory analysis, we did not find sex-related differences in brain morphometry. This analysis needs to be regarded as preliminary, because our patients were not recruited with the aim to compare brain structure between the sexes. In the recruitment process, there was no matching of male and female participants. As a result, women in our sample were significantly older and had significantly higher scores on the inattention and impulsivity subscales (Table 1). Future studies are warranted that are designed to compare clinical, psychometric, neuropsychological, and neural parameters between women and men with ADHD.

### Limitations

Our investigation has several limitations. First, in morphometric research, in particular, in patient populations, sample size is of major concern. In the recent meta-analysis by Norman et al., 27 voxel-based morphometry studies were included with a mean ADHD sample size of 34 patients (minimum: 12 patients, maximum: 131 patients; see Table 1, Supplementary Online Content) (8). Compared to these studies, our sample size of 35 women and 29 men with ADHD appears reasonable. Still, we cannot rule out the possibility that a larger sample size would have demonstrated additional associations between brain structure and ADHD symptom severity.

Second, with a mean age of 42 years in women and 38 years in men, our sample is older than the patient samples included in the meta-analysis by Norman et al. (8). A group of younger adults may have shown additional associations between brain structure (in particular, a decrease of cortical thickness and of subcortical gray matter volumes) and ADHD symptom severity, because structural abnormalities in the ADHD brain seem to diminish and finally disappear with advancing age (9, 55).

Third, we cannot compare our results to a control group of healthy individuals without ADHD. The randomized controlled trial, for which our structural brain images were acquired, contained a treatment arm and an active control arm, both of ADHD patients. Therefore, we did not recruit a healthy control group.

## Conclusion

Increased cortical thickness in the left occipital cortex may represent a mechanism to compensate for dysfunctional frontoparietal attentional networks in male adult ADHD patients. Future studies on ADHD brain structure and function should explore potential compensatory networks that might develop in adults with ADHD.

## Ethics Statement

All subjects gave written informed consent in accordance with the Declaration of Helsinki. The protocol was approved by the Ethics Committee of the Faculty of Medicine, University of Freiburg, Germany.

## Author Contributions

AP conceptualized, designed and supervised the study. MK, EH, SM, and BF were involved in the design of the study and collected the data. PS analyzed the data, prepared the figures, and wrote the manuscript. All the authors interpreted the data, provided important feedback, and revised the manuscript.

## Conflict of Interest Statement

AP has served on advisory boards, given lectures, performed phase 5 studies, or received travel grants within the last 3 years from Eli Lilly and Co., Janssen-Cilag, Lundbeck, MEDICE Arzneimittel Pütter GmbH and Co. KG, Novartis, Servier, and Shire; and has authored books and articles on psychotherapy published by Elsevier, Hogrefe, Schattauer, Kohlhammer, Karger, Schattauer, Springer, and Oxford Press. HM received speaker’s compensation from LivaNova PLC. AL declares that she received travel grants within the last year from MEDICE Arzneimittel Pütter GmbH and Co. KG and has authored articles on psychotherapy published by Elsevier and Springer.

## Footnotes

^1^https://www.isrctn.com.

^2^http://www.adhs-deutschland.de.

^3^https://surfer.nmr.mgh.harvard.edu.

^4^https://surfer.nmr.mgh.harvard.edu/fswiki/FsTutorial/GroupAnalysis.

^5^https://www.r-project.org.

## Supplementary Material

Video S1 The movie shows a left occipital area in red, in which the score on the CAARS inattention subscale predicted increased mean cortical thickness [*F*(1,27) = 26.27, *p* < 0.001, adjusted *R*^2^ = 0.4744].
