# Supplementary material for: Inattention Predicts Increased Thickness of Left Occipital Cortex in Men with Attention-Deficit/Hyperactivity Disorder
Source: Front Psychiatry. 2017 Sep 13;8:170. doi: 10.3389/fpsyt.2017.00170 (PMC5601484; doi:10.3389/fpsyt.2017.00170)
Supplement: Supplementary file 1 [file data_sheet_1.docx]

**Appendix 1:** Association between CAARS scores and subcortical gray matter volume with age as additional regressor

The Appendix lists the t values and p values of the coefficients inattention, hyperactivity and impulsivity in the linear regression model with age as additional regressor: *lm(volume ~ CAARSscore + age)*.

The Table also lists the t values and p values of the interactions between the CAARS scores and age: *lm(volume ~ CAARSscore:age)*.

* denotes: uncorrected p < 0.05; ** p < 0.01

|  | All patients (n = 64) | | Women (n = 35) | | Men (n = 29) | |
| --- | --- | --- | --- | --- | --- | --- |
|  | t value | p value | t value | p value | t value | p value |
| **inattention** |  |  |  |  |  |  |
| left caudate | 0.715 | 0.47743 | 1.604 | 0.1186 | 0.678 | 0.504 |
| left putamen | -0.592 | 0.556152 | 0.846 | 0.4039 | -0.209 | 0.83628 |
| left pallidum | -0.927 | 0.35756 | 2.742 | 0.00992 ** | -1.160 | 0.257 |
| left hippocampus | -0.219 | 0.828 | 0.087 | 0.932 | 0.780 | 0.442 |
| left amygdala | -1.417 | 0.161 | 0.379 | 0.708 | -0.212 | 0.834 |
| left accumbens | -1.891 | 0.06333 | -0.108 | 0.9149 | -1.120 | 0.2731 |
| right caudate | 0.241 | 0.81070 | 0.806 | 0.42595 | 0.266 | 0.792 |
| right putamen | -1.164 | 0.249 | 1.036 | 0.30800 | -0.857 | 0.39948 |
| right pallidum | -0.136 | 0.892505 | 1.972 | 0.05736 | -0.597 | 0.5560 |
| right hippocampus | -0.363 | 0.718 | -0.129 | 0.898 | 0.852 | 0.402 |
| right amygdala | -1.702 | 0.0939 | 0.483 | 0.632 | -1.041 | 0.308 |
| right accumbens | -0.403 | 0.68802 | 0.598 | 0.5543 | 0.123 | 0.9033 |
| **hyperactivity** |  |  |  |  |  |  |
| left caudate | 0.676 | 0.50173 | 0.433 | 0.6678 | 1.123 | 0.272 |
| left putamen | -0.246 | 0.806407 | 0.656 | 0.5165 | -0.246 | 0.80793 |
| left pallidum | -1.721 | 0.09031 | -0.101 | 0.9201 | -1.433 | 0.1638 |
| left hippocampus | -1.229 | 0.224 | -2.495 | 0.018 * | 1.350 | 0.189 |
| left amygdala | -2.331 | 0.0231 * | -2.103 | 0.0434 * | -0.167 | 0.868 |
| left accumbens | -1.655 | 0.10299 | -1.171 | 0.2501 | -0.288 | 0.7754 |
| right caudate | -0.085 | 0.93266 | 0.103 | 0.91851 | 0.140 | 0.889 |
| right putamen | -0.813 | 0.42 | 0.108 | 0.91493 | -0.462 | 0.6478 |
| right pallidum | 0.350 | 0.727556 | 0.66 | 0.51405 | 0.530 | 0.6009 |
| right hippocampus | -0.662 | 0.510 | -2.703 | 0.0109 * | 2.175 | 0.0389 * |
| right amygdala | -1.832 | 0.0718 | -1.137 | 0.264 | -0.495 | 0.625 |
| right accumbens | -0.843 | 0.40237 | -1.301 | 0.2026 | 0.655 | 0.5179 |
| **impulsivity** |  |  |  |  |  |  |
| left caudate | -0.127 | 0.89920 | -1.081 | 0.2876 | 1.743 | 0.0931 |
| left putamen | -0.582 | 0.562808 | 0.661 | 0.5134 | 0.188 | 0.85267 |
| left pallidum | -1.256 | 0.21382 | 1.063 | 0.296 | -0.925 | 0.3636 |
| left hippocampus | -2.736 | 0.00813 ** | -2.997 | 0.00523 ** | 0.134 | 0.894 |
| left amygdala | -2.573 | 0.0125 * | -1.298 | 0.203 | 0.103 | 0.919 |
| left accumbens | -2.806 | 0.00673 ** | -1.902 | 0.0663 | -0.701 | 0.4898 |
| right caudate | -0.216 | 0.83003 | -1.391 | 0.17383 | 1.393 | 0.175 |
| right putamen | -0.823 | 0.414 | 0.437 | 0.66522 | -0.049 | 0.96128 |
| right pallidum | 0.929 | 0.356420 | 1.649 | 0.10887 | 0.693 | 0.4944 |
| right hippocampus | -1.460 | 0.149 | -1.790 | 0.0828 | 0.940 | 0.356 |
| right amygdala | -1.065 | 0.291 | -0.054 | 0.957 | 0.178 | 0.860 |
| right accumbens | -1.207 | 0.2320 | -0.689 | 0.4959 | 0.206 | 0.8383 |
| **interactions** |  |  |  |  |  |  |
| inattention:age | -0.999 | 0.322 | -1.092 | 0.283 | -0.833 | 0.41264 |
| hyperactivity:age | -0.341 | 0.735 | 0.520 | 0.606 | -0.635 | 0.531286 |
| impulsivity:age | -0.254 | 0.801 | 0.799 | 0.430 | -2.052 | 0.050775 |

**Appendix 2:** Association between CAARS scores and subcortical gray matter volume with age and depression as additional regressors

The Appendix lists the t values and p values of the coefficients inattention, hyperactivity and impulsivity in the linear regression model with age and depression (BDI-II score) as additional regressors: *lm(volume ~ CAARSscore + age + depression)*.

* denotes: uncorrected p < 0.05; ** p < 0.01

|  | All patients (n = 64) | | Women (n = 35) | | Men (n = 29) | |
| --- | --- | --- | --- | --- | --- | --- |
|  | t value | p value | t value | p value | t value | p value |
| **inattention** |  |  |  |  |  |  |
| left caudate | 0.472 | 0.63880 | 1.636 | 0.1119 | 0.635 | 0.531 |
| left putamen | -0.261 | 0.794702 | 0.830 | 0.4129 | 0.149 | 0.88271 |
| left pallidum | -0.803 | 0.42494 | 2.700 | 0.0111 * | -1.386 | 0.178 |
| left hippocampus | 0.093 | 0.926 | 0.066 | 0.948 | 1.739 | 0.0943 |
| left amygdala | -1.333 | 0.188 | 0.365 | 0.718 | -0.156 | 0.877 |
| left accumbens | -2.222 | 0.030059 * | -0.122 | 0.9034 | -1.592 | 0.124 |
| right caudate | -0.211 | 0.833233 | 0.818 | 0.419868 | 0.079 | 0.938 |
| right putamen | -0.833 | 0.408 | 1.017 | 0.31715 | -0.513 | 0.61222 |
| right pallidum | -0.255 | 0.799801 | 1.958 | 0.05929 | -0.707 | 0.486 |
| right hippocampus | -0.320 | 0.750 | -0.164 | 0.8710 | 1.235 | 0.228 |
| right amygdala | -1.573 | 0.121 | 0.470 | 0.642 | -0.933 | 0.360 |
| right accumbens | -0.280 | 0.78040 | 0.587 | 0.5617 | 0.499 | 0.6220 |
| **hyperactivity** |  |  |  |  |  |  |
| left caudate | 0.572 | 0.56923 | 0.447 | 0.6582 | 1.080 | 0.291 |
| left putamen | -0.111 | 0.912312 | 0.646 | 0.5229 | -0.103 | 0.91875 |
| left pallidum | -1.657 | 0.1028 | -0.099 | 0.921 | -1.477 | 0.1523 |
| left hippocampus | -1.112 | 0.271 | -2.584 | 0.0147 * | 1.754 | 0.0917 |
| left amygdala | -2.271 | 0.0268 * | -2.101 | 0.0439 * | -0.138 | 0.891 |
| left accumbens | -1.741 | 0.08689 | -1.174 | 0.2493 | -0.411 | 0.6843 |
| right caudate | -0.263 | 0.793706 | 0.107 | 0.915616 | 0.060 | 0.953 |
| right putamen | -0.670 | 0.505 | 0.106 | 0.91632 | -0.305 | 0.76295 |
| right pallidum | 0.308 | 0.75889 | 0.659 | 0.515 | 0.507 | 0.6164 |
| right hippocampus | -0.639 | 0.525 | -2.874 | 0.00726 ** | 2.333 | 0.028 * |
| right amygdala | -1.753 | 0.0847 | -1.125 | 0.269 | -0.419 | 0.679 |
| right accumbens | -0.790 | 0.43288 | -1.324 | 0.1953 | 0.804 | 0.4291 |
| **impulsivity** |  |  |  |  |  |  |
| left caudate | -0.281 | 0.77980 | -0.889 | 0.3808 | 1.744 | 0.0934 |
| left putamen | -0.395 | 0.693922 | 0.689 | 0.4958 | 0.490 | 0.62871 |
| left pallidum | -1.176 | 0.24432 | 1.048 | 0.303 | -1.053 | 0.3025 |
| left hippocampus | -2.584 | 0.0122 * | -2.840 | 0.0079 ** | 0.736 | 0.4688 |
| left amygdala | -2.509 | 0.0148 * | -1.183 | 0.246 | 0.165 | 0.870 |
| left accumbens | -2.976 | 0.00421 ** | -1.771 | 0.0865 | -0.991 | 0.3310 |
| right caudate | -0.473 | 0.63814 | -1.192 | 0.242454 | 1.289 | 0.209 |
| right putamen | -0.624 | 0.535 | 0.475 | 0.63834 | 0.271 | 0.788 |
| right pallidum | 0.878 | 0.383326 | 1.810 | 0.0799 | 0.683 | 0.5010 |
| right hippocampus | -1.440 | 0.155 | -1.606 | 0.1183 | 1.218 | 0.235 |
| right amygdala | -0.961 | 0.341 | 0.017 | 0.986 | 0.338 | 0.738 |
| right accumbens | -1.141 | 0.25845 | -0.522 | 0.6051 | 0.496 | 0.6242 |
